# Comment on Kremer et al. Kidney Function-Dependence of Vitamin K-Status Parameters: Results from the TransplantLines Biobank and Cohort Studies. *Nutrients* 2021, *13*, 3069

**DOI:** 10.3390/nu14122439

**Published:** 2022-06-13

**Authors:** Rob Janssen, Jona Walk, Cees Vermeer

**Affiliations:** 1 Department of Pulmonary Medicine, Canisius-Wilhelmina Hospital, Weg Door Jonkerbos 100, 6532 SZ Nijmegen, The Netherlands; 2 Department of Internal Medicine, Canisius-Wilhelmina Hospital, 6532 SZ Nijmegen, The Netherlands; jona.walk@cwz.nl; 3 Cardiovascular Research Institute CARIM, Maastricht University, 6229 ER Maastricht, The Netherlands; cees.vermeer@outlook.com

In the article “Kidney Function-Dependence of Vitamin K-Status Parameters: Results from the TransplantLines Biobank and Cohort Studies”, Kremer et al. measured plasma levels of dephosphorylated-uncarboxylated matrix Gla protein (dp-ucMGP) in patients with chronic renal failure (CRF) and correlated these with plasma creatinine [1]. MGP is a vitamin K-dependent calcification inhibitor that may undergo two activation steps—phosphorylation and carboxylation—creating four MGP variants: 1. dp-ucMGP; 2. phosphorylated-carboxylated (p-c)MGP; 3. p-ucMGP; and 4. dp-cMGP [2]. Circulating dp-ucMGP reflects vitamin K status with high and low dp-ucMGP levels representing vitamin K deficiency and sufficiency, respectively [2].

Kremer et al. demonstrated that dp-ucMGP positively correlates with creatinine in CRF patients and concluded that dp-ucMGP should therefore be corrected for kidney function [1]. However, a correlation between a biomarker and kidney function does not automatically imply that it should be corrected for creatinine. Adjustment would only be appropriate if the rise is caused by a fall in renal function. Rennenberg et al., however, demonstrated that the average renal fractional extraction of MGP is independent of kidney function in hypertensive patients [3]. Theoretically, it could be the case that MGP variants in CRF are differentially excreted in urine, but Kremer et al. did not provide convincing evidence for this [1].

The correlation between dp-ucMGP and creatinine in CRF likely reflects a mechanistic link. Vascular calcification is prevalent in CRF due to disorders in mineral metabolism and increases as the glomerular filtration rate declines [4]. Calcium deposition leads to MGP upregulation to protect blood vessels from further mineralization [5]. MGP, however, is only functional after vitamin K-dependent carboxylation [6]. Activation of synthesized MGP may lead to depletion of vitamin K stores and subsequent vitamin K deficiency, which is reflected by increased dp-ucMGP levels. Adequate levels of cMGP are crucial to maintain the patency of blood vessels [5]. In a state of vitamin K deficiency, there is insufficient cMGP activity in the kidneys, exacerbating vascular deterioration and renal failure [7].

We conclude that the correlation between dp-ucMGP and creatinine is far more likely the result of vitamin K deficiency than a function of a decreased glomerular filtration rate. Regardless of any renal influence on dp-ucMGP levels, elevated dp-ucMGP levels should, in our opinion, be normalized by vitamin K administration and not through adjusting for creatinine.

## References

[1] Kremer D., Groothof D., Keyzer C.A., Eelderink C., Knobbe T.J., Post A., van Londen M., Eisenga M.F., Schurgers L.J., TransplantLines Investigators (2021). Kidney Function-Dependence of Vitamin K-Status Parameters: Results from the TransplantLines Biobank and Cohort Studies. Nutrients.

[2] Wei F.F., Trenson S., Verhamme P., Vermeer C., Staessen J.A. (2019). Vitamin K-Dependent Matrix Gla Protein as Multifaceted Protector of Vascular and Tissue Integrity. Hypertension.

[3] Rennenberg R.J., Schurgers L.J., Vermeer C., Scholte J.B., Houben A.J., de Leeuw P.W., Kroon A.A. (2008). Renal handling of matrix Gla-protein in humans with moderate to severe hypertension. Hypertens. Res..

[4] Palit S., Kendrick J. (2014). Vascular calcification in chronic kidney disease: Role of disordered mineral metabolism. Curr. Pharm. Des..

[5] Price P.A., Buckley J.R., Williamson M.K. (2001). The amino bisphosphonate ibandronate prevents vitamin D toxicity and inhibits vitamin D-induced calcification of arteries, cartilage, lungs and kidneys in rats. J. Nutr..

[6] Schurgers L.J., Cranenburg E.C., Vermeer C. (2008). Matrix Gla-protein: The calcification inhibitor in need of vitamin K. Thromb. Haemost..

[7] Roumeliotis S., Roumeliotis A., Dounousi E., Eleftheriadis T., Liakopoulos V. (2021). Vitamin K for the Treatment of Cardiovascular Disease in End-Stage Renal Disease Patients: Is there Hope?. Curr. Vasc. Pharmacol..

